# Healthcare organisation and delivery for people with dementia and comorbidity: a qualitative study exploring the views of patients, carers and professionals

**DOI:** 10.1136/bmjopen-2016-013067

**Published:** 2017-01-16

**Authors:** Frances Bunn, Anne-Marie Burn, Louise Robinson, Marie Poole, Greta Rait, Carol Brayne, Johan Schoeman, Sam Norton, Claire Goodman

**Affiliations:** 1 Centre for Research in Primary and Community Care, University of Hertfordshire, Hatfield, Hertfordshire, UK; 2 Institute for Health and Society, Newcastle University, Newcastle, UK; 3 Research Department of Primary Care and Population Health, UCL Medical School (Royal Free Campus), London, UK; 4 Department of Public Health and Primary Care, University of Cambridge, Cambridge, UK; 5 Department of Psychology, Institute of Psychiatry, King's College London, London, UK; 6 East London Foundation Trust, London, UK

**Keywords:** QUALITATIVE RESEARCH, DIABETES & ENDOCRINOLOGY, comorbidity, vision impairment

## Abstract

**Objectives:**

People living with dementia (PLWD) have a high prevalence of comorbidty. The aim of this study was to explore the impact of dementia on access to non-dementia services and identify ways of improving service delivery for this population.

**Design:**

Qualitative study involving interviews and focus groups. Thematic content analysis was informed by theories of continuity of care and access to care.

**Setting:**

Primary and secondary care in the South and North East of England.

**Participants:**

PLWD who had 1 of the following comorbidities—diabetes, stroke, vision impairment, their family carers and healthcare professionals (HCPs) in the 3 conditions.

**Results:**

We recruited 28 community-dwelling PLWD, 33 family carers and 56 HCPs. Analysis resulted in 3 overarching themes: (1) family carers facilitate access to care and continuity of care, (2) the impact of the severity and presentation of dementia on management of comorbid conditions, (3) communication and collaboration across specialities and services is not dementia aware. We found examples of good practice, but these tended to be about the behaviour of individual practitioners rather than system-based approaches; current systems may unintentionally block access to care for PLWD.

**Conclusions:**

This study suggests that, in order to improve access and continuity for PLWD and comorbidity, a significant change in the organisation of care is required which involves: coproduction of care where professionals, PLWD and family carers work in partnership; recognition of the way a patient's diagnosis of dementia affects the management of other long-term conditions; flexibility in services to ensure they are sensitive to the changing needs of PLWD and their family carers over time; and improved collaboration across specialities and organisations. Research is needed to develop interventions that support partnership working and tailoring of care for PLWD and comorbidity.

**Strengths and limitations of this study**

- Observational studies suggest that the prevalence of comorbidities in people living with dementia is high, despite this there is little research that focuses on the organisation and delivery of services for people living with dementia (PLWD) or the patient experience.
- There is evidence that in many health systems, people with dementia do not have the same access to treatment and monitoring for conditions such as vision impairment and diabetes as those with similar comorbidities but without dementia.
- This study suggests that services are not currently designed to provide appropriate integrated care for PLWD and comorbidity.
- There are immediate and simple changes that can be made to improve care for this vulnerable group such as ensuring that: the impact of a diagnosis of dementia on pre-existing conditions is incorporated into care planning, information sharing with family carers is the default option while the PLWD still has the capacity to decide, PLWD are routinely given longer appointments and dementia-specific advice is included in guidelines for conditions such as diabetes and stroke.

## Background

Dementia is primarily a condition of old age; consequently, many people living with dementia (PLWD) will have other long-term health conditions.^1^ ^2^ A UK study found that, on average, PLWD had 4.6 chronic illnesses in addition to their dementia.^3^ As the population ages, and the proportion of people with dementia and comorbidity increases, the delivery of healthcare becomes increasingly complex and challenging.^4^

Certain comorbid medical conditions may exacerbate the progression of dementia. For example, there is evidence that cognitive decline may be accelerated in older people with conditions such as type 2 diabetes or cardiovascular disease.^5–7^ Moreover, the presence of dementia may undermine a patient's abilities to access routine care, self-manage chronic conditions and engage in health maintenance activities.^8^ Despite this, healthcare systems often treat dementia as an isolated condition with little understanding of how the presence of other complex health needs might impact on processes of care, health outcomes or how this population prioritise the uptake of health services.^3^ ^9^

Navigating the different systems of care is particularly difficult for people with dementia and comorbidity, not least because they receive advice and support from different sectors of health and social care and increasingly third sector providers.^10^ ^11^ While there are policy and practice initiatives to improve healthcare for PLWD,^12^ there is little evidence of how comorbidity is experienced by PLWD and its management over time by primary and secondary healthcare services. There is, therefore, a need to consider what kind of system-based support can enable different health professionals, patients and their carers to access and manage the multiple systems of care that they need. The overall aims of this study were to explore the impact of comorbidities, for a PLWD, on access to non-dementia services and continuity of care and to identify ways of improving integration of services for this population.

## Methods

In the light of the lack of previous research in this area,^8^ we took an exploratory qualitative approach involving in-depth semistructured interviews and focus groups. The purpose was the identification and development of appropriate theory to support the development of interventions for PLWD and comorbidity and their family carers.^13^

We recruited purposive samples of PLWD and at least one of the following three conditions: diabetes, stroke or vision impairment (VI). These conditions were chosen as they are common in older people, require external monitoring and collaboration between primary and secondary care, may exacerbate the progression of dementia and their management is likely to be complicated by the presence of dementia.^14^ We also recruited family carers and healthcare professionals (HCPs) who organise and deliver care for people with stroke, diabetes and VI in primary and secondary care. Recruitment took place between December 2013 and July 2014.

PLWD were recruited via dementia registries, GP practices, memory clinics and voluntary organisations in the South and North East of England. They were asked whether they received any significant help from a family/unpaid carer, and if so, the patient's permission was sought to invite the carer for interview. HCPs were identified and recruited via specialty-specific clinical networks. The methods are reported in greater detail elsewhere.^2^

### Procedures

Interview schedules/focus group prompts were designed to explore experiences of healthcare and the barriers and facilitators to effective service provision for PLWD and a comorbid condition. They were informed by a scoping review^8^ and consultation with service user representatives, and were tailored to the type of participant and the comorbidity involved. The majority of interviews with patients involved patient–carer dyads and took place in the participants' own home, with one interview taking place in a participating memory clinic. Patient and carer interviews were conducted by one researcher (either A-MB or MP). Participants were given a copy of the study information sheet which provided contact details of the research team and a consent form, which they were asked to read and sign. They were informed that they could have a break from the interview or withdraw at any time. Five focus groups with HCPs were conducted in the clinical setting, each lasting about an hour and were facilitated by two female researchers (FB, A-MB). One interview was conducted face to face and the rest by telephone; interviews and focus groups were audio recorded and transcribed.

### Analysis

We undertook thematic content analysis^15^ which was informed by the different characteristics of continuity of care^16^ and access to care.^17^ ^18^ Data were coded independently by two researchers with emerging themes discussed with service user representatives, the project research team and advisory group. Further analysis was carried out using NVivo10 software. This paper builds on analysis of data presented elsewhere.^2^

## Results

**Figure 1:**
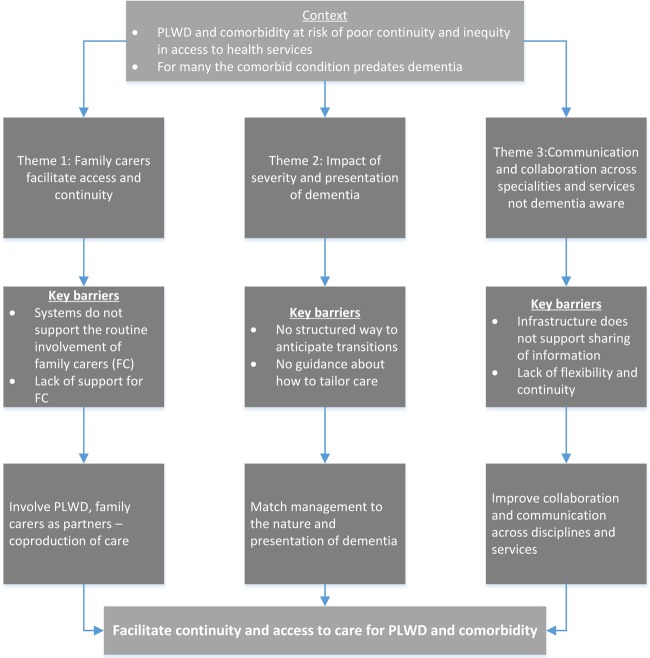
Summary of main themes showing how they link to key recommendations.

We interviewed 28 PLWD and 33 family carers (characteristics are summarised in table 1). The 56 HCPs included 10 GPs; 18 nurses (specialist and general); 13 consultants/senior clinicians specialising in stroke, diabetes and VI; 9 therapists and 2 managers. Individual interviews were conducted with 27 participants, and the rest participated in focus groups (see table 2). There are three main themes: (1) family carers facilitate access and continuity, (2) impact of severity and presentation of dementia and (3) poor communication and collaboration across disciplines and services may block access to care for PLWD. These themes and the way they link to our key recommendations can be seen in figure 1. Selected quotes supporting each theme are identified in the text and given in full in table 3.

**Table 1:** Characteristics of people with dementia (total n=28)

Type of comorbidity	Age	Sex	Ethnicity	Type of dementia	Living situation
Diabetes 31% Diabetes and VI 17% Stroke 24% VI 24% All 34%	*PLWD*—median age 82.5, range 59–94 *Carers*—median age 65, range 46–90	*PLWD* 36% female *Carers* 82% female	*PLWD*: 85% white (majority white British) *Carers*: 85% white (majority white British)	Alzheimer's disease 56% Mixed dementia 19% Vascular dementia 17% Parkinsons with dementia 8%	78% lived with a carer 64% of carers were a spouse, 14% adult child

PLWD, people living with dementia; VI, vision impairment.

**Table 2:** Details of focus groups by region

Region	Specialism	Setting	HCPs role	Number of HCPs
Midlands	Stroke	Secondary care	3 stroke consultants/1 rehab lead	4
SE	Stroke	Community	5 specialist neurological physiotherapists/1 OT	6
London	Diabetes	Community	4 specialist diabetes nurse consultants	4
SE	Diabetes	Secondary care	3 consultants/5 diabetes specialist nurses	8
East of England	VI	Secondary care	1 consultant ophthalmologist/2 orthoptists/1 specialist optometrist/1 staff nurse ophthalmology/1 senior HCA/1 intravitreal coordinator	7
Total				29

HCA, health care assistant; HCPs, healthcare professionals; OT, occupational therapist; SE, South East; VI, vision impairment.

**Table 3:** Selected quotes illustrating the themes

Quote	Theme 1: Coproducing care with PLWD and family carers
1	as a family member you're the person who knows that person better than anyone else so you can see when it's not, when it's not right, when it's going wrong—Carer Diabetes 4* SE
	it was like when she had her cataract done, I actually went into the room with her… you know, because one nurse kind of looked at me and she said ‘no, if you wait in the waiting room’, I went ‘well, no—my sister has a memory problem so I'll have to stay’—Carer Diabetes/VI 6 SE
2	her feet were black and I was concerned, because we've got, in the paternal side of my family, she's got aunts and her mother was blind, aunt had amputation of the toes—Carer Diabetes/VI 3 SE
3	and now I go with him for all his appointments…I have got a notebook there which I use to note everything, you know, when it started [sound of paper rustling] for myself, for my own, you know…I used to record everything, ‘seen by so and so, what prescribe and when to be seen again’ and all these things.—Carer diabetes/VI 2 SE
4	you see one person one time and then you'd have, tell them what they need to know and then you see the next person and they don't know, do they. You have to go all through it yeah, you have to start again. But I mean, that actually is a problem with the NHS all the way through, I mean, because it's a kind of, you know, you're not always treated as a whole person, you're treated as individual bits, aren't you—PLWD and Carer VI 7 SE
5	the greatest difficulty is when that individual lives alone and doesn't have an able partner, because then their care can become very disjointed or they're not, they're not able, often they, an appointment's made or they, and they won't answer the door or they forget and so it's when somebody's on their own that you have the biggest issues and lack of joined up care—Physiotherapist 1 SE
6	do you remember that mum, you know your method for testing your blood that you'd used for years, last Easter the nurse came on Maundy Thursday, the day before Easter and she gave you a new machine to do it…And you could not fathom it at all…No, no, none of us could, could we? It was chaos…—Carer Diabetes 4 SE
7	gradually I took over the medication, each step was really painful, you know ‘cos he always used, he was on by the time when he started sort of losing grip on things he was on a lot of medication, six or eight different pills a day and he would line them up and take them one at a time and so on, and then I started putting them in dosette boxes and then he started not remembering to take them and then he would take them at random so gradually I took over the whole thing and I mean there were a lot of tears and agony—Carer Stroke 7 SE
8	I know yesterday you had a bit of a problem because you thought, when I phoned you up in mid-morning you thought that the lady hadn't been to give you your medications and your Cornflakes but in fact she had, hadn't she? (Carer Diabetes 4 SE) she had, yeah. (PLWD diabetes 4 SE) so mum ended up having two breakfasts yesterday—Carer and PLWD diabetes 4 SE
9	the Alzheimer's Society have been fantastic…Oh the Alzheimer's Society, oh .. that's a godsend that is, absolutely godsend, yeah—Carer Stroke 4 SE
10	they have a diabetic nurse and she rings up every now and again to get her readings I don't think that's very good, that's one of the services that I don't think is very good to be honest.*—*Carers Diabetes 7 SE
11	[GP] yes, now she's gone ahead with loads of things because she says ‘are you getting this, are you getting that,’ we told her what we'd had and what you know what he doesn't have, so she says ‘right I shall get in touch with these people’ she said ‘and help you’. Now as it happens she must have done very quickly, because we had a lady from the social services yesterday—Carer Diabetes NE
12	in fact when I know that I've got one of my patients with dementia booked in I will ask, I will send an email in advance to the administrator, to the receptionist to sort of call them on the day to remind them—GP 2 London
Theme 2: Matching management to the nature and presentation of dementia	
13	we had a timer at the beginning and it bleeped when he should take a tablet, well he would go and turn the bleeper off and forget to take the tablet so—Carer VI 6 SE
14	another risk that was highlighted to me recently was a patient in this circumstance who was previously self-managing, District Nurses had to take over, but the insulin has to stay in-house and the nurses don't carry it around, so this patient was, it transpired this patient was given her own insulin and the District Nurses were coming in after and administering again, it took a while to establish that—Diabetes Consultant 2 FG1 SE
15	I think as you get more experienced, it's quite a difficult decision but as you get more experienced your decision changes. I'm certainly quite…personally, I don't know how others are but I certainly am quite aggressive about cataract surgery in people with dementia, I think that it's got a very low downside, the chances of something going wrong are very remote and if you make it work and you make them function better then fine.—Ophthalmologist 1 London
16	I wouldn't refer someone who was uncooperative … I have had a patient who got up in the middle of a cataract operation and refused to have anything further done and lost the vision in his eye—GP 4 SE
17	just training really, just I think we just need that extra training just to, in this particular aspect, clinical and awareness of what to do—Senior Orthoptist, VI focus group, East of England
Theme 3: Working across disciplines and organisations	
18	I think new services like in L1 [London Borough] we have the community matrons have actually been of great help because they are more of care co-ordinators which I think do help these people with comorbidities in the community—GP 2, London
19	but obviously anywhere new that we go, like for this colonoscopy and all that sort of thing, I always mention, you know, ‘he has dementia quite, quite severe dementia’, I think when we went for a blood test for this colonoscopy it wasn't on his notes there, although it was on the original colonoscopy referral sort of thing. So it seems that within the hospital setup they don't always transfer all relevant information between departments—Carer Diabetes 1 SE
20	and I think that's a key point I was going to make is one of the big stumbling blocks we have is the fact that services or parts of different Trusts so the Mental Health Services sit within the H Partnership Trust so they don't use the same system as us so we can't share notes, the GPs use a different system again so it makes it very difficult to communicate to even find out what services people are under, you know, if that could be improved, if we could all be on the same system that would be good [laughs]—Physiotherapist FG SE
21	memory loss, no, they're not interested in that, they're interested in treating the symptoms of diabetes not somebody else's, it's almost like somebody else's problem but I don't mean that hard heartedly, I mean that we are dealing with this bit, there's nobody, other than my GP looking at the whole picture—PLWD Diabetes/Stroke/VI 1 SE
22	but if you're reliant on District Nurses for example who got their own, you know, they've got their timetable of what they need to do in their work to get through, and they have to administer it at a set time and that can be incredibly disruptive to the individual—Diabetes Consultant 2 FG1 SE
23	then just to simple tie up the medication and monitoring of diabetes with the provision of meals is basically all that needs to be done. And I think that's where it falls apart a lot of the time because people who can't self-manage will often be reliant on a district nurse or a community nurse to perhaps come in and oversee the medication or give them their Insulin, but they won't be responsible for ensuring that that person has their breakfast or, so you get big gaps between one and the other and that really is not helpful. And that's how people do end up having falls and being admitted to hospital, yeah—Diabetes Consultant FG1 SE

*Each interview was given a unique number.

FG, focus group; GP, general practitioner; NE, North East; PLWD, people living with dementia; SE, South East; VI, vision impairment.

### Theme 1: family carers facilitate access and continuity

Family members were often proactive in facilitating continuity and negotiating access to services for their relatives with dementia. This included acting as an advocate for their family member with dementia, noticing when something was wrong and seeking help (Q1, Q2), and helping clinicians make treatment decisions, such as whether to thrombolyse a PLWD after a stroke. Family carers also had a significant role in coordinating their relative's care, navigating healthcare systems and facilitating continuity of care; for example, managing appointments, organising transport, keeping records of test results and medication (Q3) and actively transferring information between HCPs and different services (Q4). The availability of a family carer to act as a proxy, and provide consent, information and postdischarge support impacted on a PLWD's access to care. HCPs recognised that PLWD who lived alone, or did not have support from a family carer or advocate, were particularly vulnerable and may have poorer access to care (Q5).

Although HCPs in our study valued the role family carers played, there was little formal recognition of the carers' role, and no systems for negotiating how or when carers' views could be incorporated into care planning. This was reflected in the many examples provided by our interviews where carers felt undervalued or excluded from decision-making about their relative's care (Q6). HCPs' concerns about confidentiality meant that carers sometimes had trouble accessing the information they needed to manage their relative's care. For example, being refused copies of letters or details of hospital appointments. Although a number of carers and PLWD mentioned lasting power of attorney, this was seen as facilitating management of financial affairs rather than healthcare.

There were many challenges for family carers. These included difficulty in understanding how health systems worked and who to contact, their own health problems, emotional and practical challenges of changing roles (Q7) and living at a distance and/or with work and family commitments that made taking on responsibilities for day-to-day care difficult. Caring at a distance may be particularly problematic for carers of PLWD as it is difficult for them to offer support or to monitor adherence to medication over the phone (Q8). Support from social networks, such as extended family, friends and religious groups, and from third sector providers (Q9) were clearly important to PLWD and their carers, but formal support from health and social care was often seen as inadequate (Q10).

PLWD and family carers valued continuity, in terms of relationships with practitioners but also in terms of encounters that factored in the impact of dementia, that built on earlier conversations and appointments and that included people with dementia and their carers in decision-making. Many PLWD and carers reported positive relationships with their GPs and recognised the role that GPs played in coordinating care (Q11). HCPs cited the use of practices for mitigating the impact of living with dementia such as reminding patients of upcoming appointments, giving them longer appointments (Q12) or making sure that it was always the same HCP that saw patient and carer. However, these practices were at the discretion of individual HCPs and not formal processes of care.

### Theme 2: impact of severity and presentation of dementia on management

**Figure 2:**
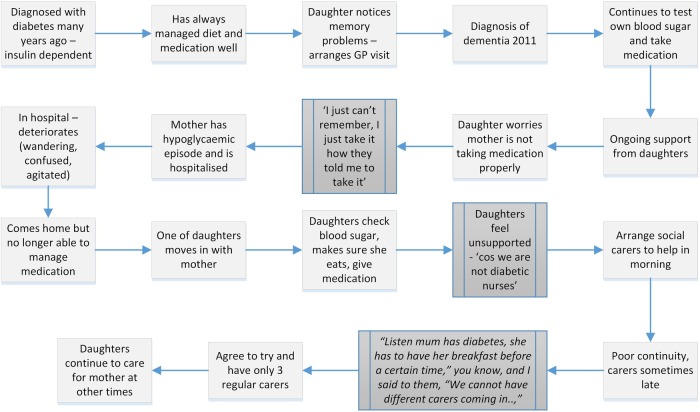
Illustration of one person's transition from self-management to dependence.

How PLWD managed their care, for example, either independently, in tandem with a family carer or with external health and social care support, was linked to where they were on the dementia trajectory. Some people with early-stage dementia were still able to self-manage their care. As the dementia got worse, the PLWD's ability to self-manage declined and responsibility moved, either partly or totally, from the PLWD to a carer. These transitions often happened when strategies to facilitate self-management, for example, memory aids, diaries and dosette boxes, ceased to be effective (Q13). An example of such a transition, which resulted in a hypoglycaemic attack and hospitalisation, can be seen in figure 2. Data showed that HCPs did not have a structured way of anticipating changing needs and these were times when PLWD could drop out of the system (such as failure to keep appointments), be at risk of exacerbation (eg, taking too much or too little medication) (Q14) or family breakdown in care arrangements that had previously worked well.

The data show how the severity of dementia, and the ability of the PLWD to cooperate with treatment, impact on clinical decision-making. Access to care was also affected by clinician's previous experiences and their attitudes towards risk. For example, there were contrasting attitudes towards the benefits of performing cataract surgery for PLWD and different opinions about the safety of taking someone off insulin (Q15, Q16). There was also considerable variation in access to care such as screening and monitoring for diabetes.

HCPs in all specialities spoke about the importance of personalising care for PWLD. For example, personalising target values for blood glucose for people with diabetes, simplifying medication regimes for people with VI and adapting stroke rehabilitation programmes. However, there was little evidence of knowledge about personalising care for PLWD being widely translated into practice and many HCPs appeared to lack the skills and confidence to tailor investigations and care to the needs of PLWD, for example, the creation of cognitive impairment friendly eye tests (Q17). This was reflected in the many examples provided by PLWD and their family carers where they felt HCPs had not recognised their need for a different approach. For example, giving patients complicated medication regimes they were unable to manage (Q6).

### Theme 3: communication and collaboration across specialities and services is not dementia aware and may block access to care

Most of our participants with dementia and comorbidity had input from a variety of services and specialties. While HCPs gave some examples of successful integrated working (Q18), such as community matrons, or a community multidisciplinary team, a number also outlined how current infrastructure did not support the sharing of information across different specialities (Q19), particularly across mental and physical health services (Q20). HCPs were often unaware that someone had a diagnosis of dementia which impacted on their ability to carry out assessments. For example, VI specialists, who relied on patients being able to provide feedback from vision tests, reported that they were frequently not made aware a patient had dementia before they saw them in clinic.

For many of our participants, their comorbid health condition predated the diagnosis of dementia. Despite this, there appeared to be inadequate consideration by some services of the implications of a diagnosis of dementia on the management of existing conditions (Q21). HCPs from all specialities suggested ways of helping patients navigate multiple encounters with different professionals. This included key workers, case managers or hand-held patient records. However, while this indicated a recognition of the need to bridge the gap between different services, there were few examples of such interventions in practice.

A lack of flexibility in health and social care was also a problem. For example, HCPs reported that in some instances, insulin regimens had to be altered to fit with the schedules of district nurses who were only available at certain times of the day (Q22). The split between social care and healthcare was also identified as a particular problem. Supporting PLWD to live independently at home is invariably seen as social care. This broke down when social services carers were not able to test blood sugars or oversee medication for people with diabetes, making it difficult to coordinate meals and medication and putting PLWD at risk of hypoglycaemia (Q23).

## Discussion

We conducted interviews with 28 people with dementia and 33 family carers, and focus groups or interviews with 56 HCPs. What emerged from our analysis is that in order to facilitate access to care and improve continuity for PLWD and comorbid conditions, there is a need for: coproduction of care in which HCPs, PLWD and family carers work in partnership, the matching of management to the needs of the individual (including ways of anticipating changes in needs and tailoring care appropriately), and improved collaboration across specialities and organisations. We found examples of good practice, but these tended to be about the behaviour of individual practitioners rather than system-based approaches; current systems may unintentionally block access to care for PLWD. Our study further highlights not only how family carers are often responsible for negotiating continuity and access for family members with dementia but also how care systems often hinder rather than support their efforts.

While qualitative research does not generally set out to be representative, it is appropriate to consider the transferability of findings. Our study was conducted in two, primarily urban, geographical areas in England, and participants in other areas or countries may face different barriers to accessing services. However, previous reviews have found many similarities in the experiences of people with dementia and their family carers regardless of culture, context or country.^8^ ^19^ Our findings, therefore, should have resonance for the wider international community of older people with dementia and comorbidity. HCPs in our study were a self-selecting sample willing to have their practice examined. As such, it is possible that they may have had more awareness of the needs of PLWD and more interest in their care needs than their colleagues. However, the accounts of HCPs were validated against the accounts of PLWD and their family carers. Like much qualitative research with people with dementia,^19^ the majority (78%) of our participants with dementia lived with a family carer and we are able to say less about the experiences of those who live alone.

Like previous research, we found that fragmented care, clinical guidelines that focus on single conditions and poor communication and collaboration between different specialities were barriers to continuity and access to care for PLWD.^3^ ^20^ Models of care designed to improve interprofessional working include components such as case management, specialist nursing support, comprehensive geriatric assessment and colocation of different specialities to promote integration and holistic care.^21^ Randomised controlled trials have provided conflicting information about the clinical or economic benefits of many of these interventions for PLWD,^22–25^ although non-randomised studies have found positive impacts, particularly on patient and caregiver satisfaction.^26^ ^27^ Our study suggests that relatively minor changes to healthcare systems, such as ensuring that PLWD are identified in advance of visits to outpatient services and primary care, or for providers to make information sharing with family carers the default option while the person still has capacity to decide, could lead to improvements in care.

PLWD are often reliant on others, typically family members, to act as their advocate or help with care management. There is a need for approaches to care that recognise that families are often crucial allies for quality and safety and should, subject to patient agreement, be routinely involved in decision-making for PLWD and comorbidity.^28^ Such approaches, however, need to incorporate consideration of the capacity of patients and their family carers to attend to current and future healthcare demands, and the support needs of the family carer.^29^ Participants in our study ranged from those who were able to self-manage their condition and navigate health and social care systems with minimal support to those who required extensive support, often provided by family carers. Consideration should be given to the recognition and management of times of transition, such as when worsening symptoms of dementia, or a medical emergency, impact on a PLWD ability to undertake appropriate self-management. PLWD and their family carers need for support may be particularly acute at such times. People with dementia who live alone or do not have family support may need additional help to navigate systems and access care.

Our study supports calls for health and social care services to take a collaborative approach that recognises PLWD and family carers as partners in their care.^30^ However, such approaches may be difficult to embed in care for PLWD where decision-making is complicated by concerns about polypharmacy, consent, concordance and the appropriateness of treatment in people with advanced dementia. Further research is needed to develop interventions that support partnership working and that incorporate the consideration of the risk–benefit balance of different treatment options. The call for healthcare for PLWD to be individualised is not new. Despite this, our study suggests that more research is needed to identify how assessment, treatment and ongoing support for conditions such as diabetes can be tailored to meet the needs of PLWD and that is responsive to the way in which changes in the severity and symptoms of dementia might impact on the support required. For example, the development of appropriate methods of assessment for vision in PLWD.

## Conclusions

Significant numbers of PLWD have comorbid conditions such as stroke, diabetes and VI; and many of them have multimorbidity. The presence of dementia complicates the delivery of healthcare, and magnifies the known difficulties people with long-term conditions experience when navigating health and social care. Current approaches to improve dementia awareness in the workforce are unlikely to address the challenges described in the study. There is a need for changes in the way PLWD are integrated into systems of care. The delivery of high-quality care to PLWD demands a particularly high standard of care across multiple domains, including communication, multidisciplinary care and clinical decision-making.^31^ Key elements include: the PLWD and family carer at the centre,^32^ ^33^ flexibility around processes, good communication between services, ensuring that all services are aware when someone has a diagnosis of dementia, taking into account the impact of the nature and presentation of dementia on pre-existing conditions, and incorporating this into guidelines and care planning.
